# Comparison of paravertebral block vs. general anesthesia for percutaneous nephrolithotomy: A retrospective study

**DOI:** 10.3389/fmed.2023.1081530

**Published:** 2023-02-03

**Authors:** Miaomiao Fei, Wendong Qin, Guanghui An, Dujian Li, Cheng Li, Lize Xiong

**Affiliations:** ^1^Department of Anesthesiology and Perioperative Medicine, Shanghai Fourth People’s Hospital, School of Medicine, Tongji University, Shanghai, China; ^2^Shanghai Key Laboratory of Anesthesiology and Brain Functional Modulation, Shanghai, China; ^3^Translational Research Institute of Brain and Brain-Like Intelligence, Shanghai Fourth People’s Hospital, School of Medicine, Tongji University, Shanghai, China; ^4^Clinical Research Center for Anesthesiology and Perioperative Medicine, Tongji University, Shanghai, China; ^5^Department of Urology, Shanghai Fourth People’s Hospital, School of Medicine, Tongji University, Shanghai, China

**Keywords:** paravertebral block (PVB), general anesthesia (GA), percutaneous nephrolithotomy (PCNL), postoperative analgesia, the visual analog scale (VAS) pain score

## Abstract

**Background:**

General anesthesia is used in the majority of patients undergoing percutaneous nephrolithotomy. To reduce the general anesthesia-related risks and complications, this study evaluated the efficacy and safety of the paravertebral block as a novel and alternative anesthetic method for percutaneous nephrolithotomy.

**Methods:**

This was a retrospective study. A total of 198 patients under percutaneous nephrolithotomy were included. Among them, 76 patients received paravertebral block and 122 received general anesthesia. Patients’ characteristics, surgical outcomes, anesthetic outcomes, and perioperative complications and the visual analog scale (VAS) were recorded to evaluate the efficacy and safety of paravertebral block compared with general anesthesia. Intergroup differences of the parameters were analyzed using an independent *t*-test and χ^2^-tests appropriate.

**Results:**

Seventy-six patients who underwent paravertebral block completed the surgery successfully, three patients were supplemented with propofol for discomfort during ureteroscopy, and two patients were supplemented with remifentanil for incomplete nerve blockade. Patients who underwent paravertebral block had a higher American Society of Anesthesiologists grade and heart function grade, including patients with contraindications to general anesthesia. Intraoperative and postoperative adverse events and the anesthesia costs were less in patients who underwent paravertebral block. VAS pain scores during the postoperative period in patients who underwent paravertebral block were lower than those in patients who underwent general anesthesia without the use of patient-controlled intravenous analgesia.

**Conclusion:**

In this retrospective study, paravertebral block was found to be effective and safe in providing intraoperative anesthesia for percutaneous nephrolithotomy, and had less adverse events and anesthesia costs. Paravertebral block is an attractive alternative anesthesia for patients at increased risk of comorbidities following general or neuraxial anesthesia.

## Introduction

Urolithiasis is one of the most common disorders among urinary diseases (1). A review of recent epidemiological studies indicated that the prevalence of urolithiasis is more than 10% (2), and that the recurrence rate approaches 50% after 10 years (3, 4). Approximately 70% of the population affected by this disorder is between 20 and 50 years old. One of the major symptoms is renal colic, a sudden intense flank pain (5). Nephrolithiasis, a subtype of urolithiasis, affects millions of people each year in China (6), and this high prevalence is associated with frequent surgical interventions (7, 8). Percutaneous nephrolithotomy is a widely used surgical procedure for the elimination of large and complex upper renal calculi (9, 10), and is the gold standard for treating nephrolithiasis with fewer complications than open surgery (11, 12). However, even though percutaneous nephrolithotomy is a minimally invasive procedure, the intra- and postoperative pain perceived by patients is intense (13, 14). General and neuraxial anesthesia are commonly used for percutaneous nephrolithotomy (15, 16). However, they are often associated with an increased risk of complications or are contraindicated (17), especially in the elderly or patients with multiple comorbidities.

The paravertebral block is a technique involving the injection of a local anesthetic adjacent to the intervertebral foramina where the spinal nerves exit the thoracic vertebral canal, resulting in ipsilateral segmental sympathetic nerve blockade (18–20). Paravertebral block is a simple and effective technique for unilateral procedures, with minimal incidence of hypotension and urinary retention (7, 21). There are several reports on the use of paravertebral block for percutaneous nephrolithotomy, though it is mainly used for intra- and postoperative analgesia (13, 21–23). Mei et al. presented reports to share their experience with paravertebral block as the main anesthesia used for percutaneous nephrolithotomy (24, 25). Other studies compared paravertebral block combined with moderate sedation with intraspinal anesthesia for percutaneous nephrolithotomy (26). These demonstrate the effectiveness of paravertebral block. However, reports about the application of paravertebral block for anesthesia in percutaneous nephrolithotomy are still few, and available data are limited. Thus, more studies are needed to explore the efficacy and safety of the paravertebral block for percutaneous nephrolithotomy. In the present retrospective study, we aimed to evaluate the effects of ultrasound-guided six-segment paravertebral block to provide anesthesia for percutaneous nephrolithotomy.

## Materials and methods

This study was approved by the Institutional Research Ethics Committee of the Shanghai Fourth People’s Hospital (approval number: 202011115-001). As a retrospective study, that is, a re-exploration and utilization of past data, the Ethics Committee approved our waiver of informed consent.

### Patients

This retrospective study was conducted on patients who attended the Shanghai Fourth People’s Hospital from January 2019 to October 2020. All data were obtained from the anesthesia system, the anesthesia record, and the postoperative visit record. A total of 198 patients who had large or complex renal calculi and underwent percutaneous nephrolithotomy were included in the study. The patients were assigned to the paravertebral block group (PVB group) or the general anesthesia group (GA group) according to the anesthesia type they received. Seventy-six of the patients underwent paravertebral block and 122 received general anesthesia. Moreover, the patients in the GA group were further divided into two subgroups based on whether a patient-controlled intravenous analgesia (PCIA) was not used (GA-1) or used (GA-2 group). Age, sex, body mass index, and comorbidities were recorded, including hypertension, diabetes, chronic obstructive pulmonary disease, chronic renal failure, and coronary heart disease.

### Paravertebral block

**FIGURE 1:**
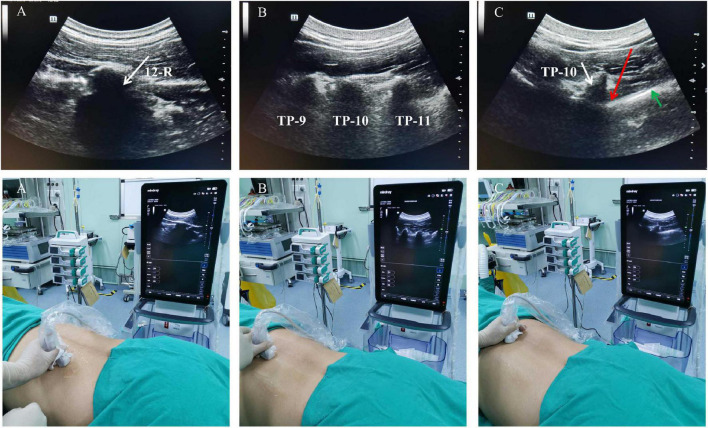
Illustration of the step-wise technique for paravertebral block. **(A)** The probe is moved cephalad to determine the position of the 12th (white arrow). **(B)** The probe is rotated to the sagittal plane and the transverse process of T_9_, T_10_, and T_11_ is in the same visual field. **(C)** The probe is moved to determine the paravertebral spaces (red arrow), transverse process of T_10_ and the pleura (green arrow) is identified. R, rib; TP, transverse process.

The patients were positioned in the prone position, and standard monitoring was performed with non-invasive blood pressure measurement, electrocardiography, and pulse oximetry. The ultrasound-guided paravertebral block was performed using a linear array 5–10 MHz probe (Sonosite, Bothell, WA, USA). The probe was positioned on the lumbar spine, parallel to the ribs. The probe was moved cephalad until the 12th rib was visualized. Then, the probe was rotated anticlockwise for a standard sagittal slice of the rib. The probe was moved medially to identify the T_11_ and T_12_ transverse processes. The T_8_-L_1_ transverse processes were also identified by moving the probe cephalad and caudad. The T_8_-L_1_ paravertebral spaces were chosen for the procedure, and 5 mL of 0.33% ropivacaine was intermittently injected at each segment, the total dose of ropivacaine was 100 mg (Figure 1). The blocking effect was evaluated 15 min later, and based on the patient’s condition, additional sedatives or analgesics were administered or not. After a successful block, lidocaine gel was injected into the urethra for 2 min before ureteroscopy. Surgery was performed after the patient’s pain was resolved.

### General anesthesia

The patients induced by sufentanil (0.3 μg/kg), propofol (2 mg/kg), and cisatracurium (0.2 mg/kg), and dexmedetomidine (1 μg/kg) was administered *via* intravenous pump; tracheal intubation was performed 2 min later. General anesthesia was maintained with propofol (5 mg/kg/h) and remifentanil (10 μg/kg/h). Anesthesia of patient was monitored using the standard anesthetic observation for changes in vital signs, such as heart rate, blood pressure, respiratory effort, EtCO_2_, and body temperature in response to surgical stimulation. PCIA was provided with sufentanil (2 μg/kg) and butorphanol (1 mg/kg) to 150 mL at the end of surgery.

### Surgical procedure

Swabs were used to test the effect of blocking ipsilateral T_8_ to L_1_ region anesthesia 15 min later, surgery was performed after the patient’s pain was resolved. Percutaneous nephrolithotomy was performed in two stages: first, the patient was placed in the lithotomy position, ureteroscopy was performed, and the ureteral and urinary catheters were indwelled. Then, the patient was moved into the prone position, and after ultrasonic positioning, nephroscopy and lithotripsy were performed for percutaneous nephrolithotomy.

### Evaluation of the effects and safety of paravertebral block and general anesthesia

The American Society of Anesthesiologists (ASA) grade and heart function grade according to the New York Heart Association (NYHA) were recorded to evaluate the tolerance to paravertebral block and general anesthesia in different patients, especially the elderly and patients in poor general condition. Our primary outcome was operative pain as measured by the visual analog scale (VAS, 0–10; 0: no pain, 10: maximum pain) in patients who received the paravertebral block. The secondary outcomes include intra- or postoperative adverse events, such as hypoxia (SPO_2_ < 90), hypertension (MAP more than 20 percent above baseline), hypotension (MAP less than 20 percent above baseline), postoperative itching, nausea, and vomiting, were recorded to assess the safety of the two types of anesthesia. Moreover, the satisfaction with anesthesia among surgeons and patients, the number of patients who required additional sedation and analgesia, including propofol, and remifentanil, was recorded. Hospitalization days and procedural costs were also analyzed to compare the economic factors between the two types of anesthesia. Other data, including anesthesia duration, the volume of fluid infused, and surgical data, were also recorded and compared.

### Statistical analysis

All data are presented as medians, means, or incidence, as appropriate. The categorical and continuous variables were analyzed using χ^2^-tests and independent *t*-tests, respectively. Microsoft Excel 2013 (Microsoft Corp., Redmond, WA, USA) was used to record the data, and the analyses were performed using SPSS Statistics for Windows, version 22 (IBM Corp., Armonk, NY, USA). *P* < 0.05 indicated statistical significance.

## Results

A total of 198 patients were included in this study. Among them, 76 patients were anesthetized with paravertebral block and 122 received general anesthesia. The patients’ characteristics are summarized in Table 1. There were no significant differences in sex, age, weight, height, and body mass index between groups. The PVB group had significantly higher ASA (*P* = 0.005) and NYHA heart function grades (*P* = 0.001). There were no significant between-group differences in comorbidities, except for hypertension, which was more frequent in the GA group (*P* = 0.03).

**TABLE 1:** Characteristics of included patients.

	PVB group (*n* = 76)	GA group (*n* = 122)	*P*-value
Gender (males/females)	68/8	108/14	0.84
Age (years)	62.86 ± 11.41	60.48 ± 11.23	0.98
Weight (kg)	75.14 ± 10.22	74.97 ± 9.94	0.99
Height (cm)	1.69 ± 0.55	1.70 ± 0.52	0.88
BMI	26.25 ± 3.04	26.04 ± 3.18	0.78
ASA grade (I/II/III/IV)	3/58/11/4	19/91/12/0	0.01
Heart function grade of NYHA (I/II/III/IV)	4/56/16/0	27/84/11/0	<0.01
**Comorbidity**			
Hypertension	53 (69.7%)	66 (54.1%)	0.03
Diabetes	45 (59.2%)	58 (47.5%)	0.11
COPD	10 (13.2%)	13 (10.7%)	0.59
CRF	3 (3.9%)	3 (2.5%)	0.55
CHD	17 (22.4%)	20 (16.4%)	0.29
The history of stroke	7 (9.2%)	6 (4.9%)	0.24

Values are given as *n* (%) or means ± SD. PVB group, paravertebral block group; GA group, general anesthesia group; BMI, body mass index; COPD, chronic obstructive pulmonary disease; CRF, chronic renal failure; CHD, coronary heart disease.

The clinical parameters of surgery are summarized in Table 2. There were no significant differences regarding the duration of surgery between groups. Surgical complications and surgeon satisfaction with anesthesia did not differ between groups (*P* > 0.05). Among the 76 patients who underwent paravertebral block, three received sedation during ureteroscopy and five in feeble conditions required assistance with repositioning during the procedure.

**TABLE 2:** Clinical parameters of surgery.

	PVB group (*n* = 76)	GA group (*n* = 122)	*P*-value
Duration of Surgery (min)	75.79 ± 8.94	77.38 ± 10.39	0.27
Duration of ureteroscopy (min)	14.42 ± 2.62	14.18 ± 2.39	0.51
Duration of PCNL (min)	61.37 ± 9.38	63.20 ± 10.42	0.24
**Surgical complications**			
Pneumothorax	0 (0)	0 (0)	1.00
Bleed	0 (0)	1 (0.8%)	0.43
Infection	1 (1.3%)	1 (0.8%)	0.73
Organ injury	0 (0)	0 (0)	1.00
Self-positioning without assistance	68 (89.5%)	0 (0)	<0.01
Surgeon satisfaction with anesthesia (0–10 score)	9.37 ± 1.08	9.35 ± 0.97	0.91

Values are given as *n* (%) or means ± SD. PVB group, paravertebral block group; GA group, general anesthesia group.

The anesthesia parameters of the patients are shown in Table 3. The anesthesia duration was longer in patients who underwent paravertebral block than that of patients receiving general anesthesia (99.41 ± 7.79 vs. 88.93 ± 11.80 min, *P* < 0.001). The fluid infusion volume was less in the PVB group (785.52 ± 99.60 vs. 1,045.08 ± 151.22 mL, *P* = 0.007). The VAS pain score during the procedure in the PVB group was 1.49 ± 0.90; however, this value could not be compared between groups since patients under general anesthesia could not provide this score. Three patients were supplemented with propofol (1 mg/kg, intravenous injection) because of discomfort during ureteroscopy and 2 patients were supplemented with remifentanil (2 μg/kg/h, intravenous pump) for incomplete nerve blockade. All patients in the GA group also received these medicines. Intraoperative adverse events such as hypotension were less frequent in patients with paravertebral block (*P* = 0.01), and there were no between-group differences in hypertension and hypoxia.

**TABLE 3:** Anesthesia parameters of the patients.

	PVB group (*n* = 76)	GA group (*n* = 122)	*P*-value
Duration of anesthesia, min	99.41 ± 7.79	88.93 ± 11.80	<0.01
VAS during PCNL	1.49 ± 0.90	–	–
**Usage of sedative and analgesic drugs**			
Propofol	3 (3.9%)	122 (100%)	<0.01
Remifentanil	2 (2.6%)	122 (100%)	<0.01
Volume of fluid infused, ml	785.52 ± 99.60	1,045.08 ± 151.22	<0.01
**Intraoperative adverse events**			
Hypoxia	1 (1.3%)	0 (0%)	0.81
Hypertension	10 (13.2%)	13 (10.7%)	0.59
Hypotension	1 (1.3%)	13 (10.7%)	0.01

Values are given as *n* (%) or means ± SD. PVB group, paravertebral block group; GA group, general anesthesia group. Hypoxia is defined as SPO_2_ < 90, Hypertension is defined as MAP more than 20 percent above baseline, Hypotension is defined as MAP lower than 20 percent above baseline.

The GA group was subdivided according to the use of PCIA; there were 68 patients in the GA-1 group (general anesthesia without PCIA) and 54 patients in the GA-2 (general anesthesia with PCIA) group. Table 4 shows the postoperative data of the three groups. The patients in the PVB group had a lower incidence of postoperative itching, nausea and vomiting (*P* = 0.01); PCIA had no significant effect on these symptoms in patients with general anesthesia. No significant differences emerged in hospitalization and patient satisfaction. The anesthesia cost was lower for paravertebral block than that of general anesthesia, without or with PCIA (93.58 ± 27.25 vs. 278.89 ± 29.08 and 387.39 ± 20.44 $, respectively; *P* < 0.001).

**TABLE 4:** Postoperative complications and other related indicators.

	PVB group (*n* = 76)	GA group (*n* = 122)		*P*-value
		**GA 1 group (*n* = 68)**	**GA 2 group (*n* = 54)**	
PONV	1 (1.3%)	7 (10.3%)	9 (16.7%)	0.01
Itching	0 (0)	1 (1.5%)	2 (3.7%)	0.23
Postoperative analgesia	0 (0)	0 (0)	54 (100%)	<0.01
Hospitalization, days	7.09 ± 0.61	7.06 ± 0.79	7.18 ± 0.68	0.59
Anesthesia cost, $	93.58 ± 27.25	278.89 ± 29.08	387.39 ± 20.44	<0.01
Patient’s satisfaction with anesthesia (0–10)	8.78 ± 1.11	8.81 ± 0.97	8.53 ± 1.00	0.30

Values are given as *n* (%) or means ± SD. PVB group, paravertebral block group; GA 1 group, general anesthesia without PICA Group; GA 2 group, general anesthesia with PICA Group; PONV, postoperative nausea and vomiting.

**FIGURE 2:**
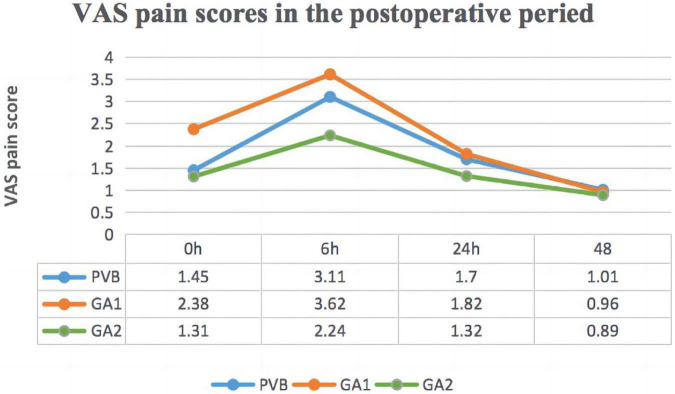
The visual analog scale (VAS) pain scores in the postoperative period. PVB group, paravertebral block group; GA 1 group, general anesthesia without PICA Group; GA 2 group, general anesthesia with PICA Group.

The PVB group did not receive postoperative analgesia, we compared the VAS scores among the three groups in the postoperative period in Figure 2. The mean VAS scores in the PVB and GA-2 group at the end of the surgery were not significantly different; however, they were both lower than those in the GA-1 group (*P* < 0.001 and *P* = 0.01, respectively). Six hours after the procedure, the mean VAS score in the PVB group was higher than that in the GA-2 group and lower than that in the GA-1 group (*P* < 0.001 and *P* < 0.001, respectively). At the 24-h time point, the difference between the PVB and the GA-1 group was not significant; patients who received PCIA had a significantly lower VAS score than those in the other two groups (*P* = 0.047 and *P* = 0.001, respectively). The mean VAS pain score differences among the three groups were not statistically significant at the 48-h time point.

## Discussion

In this retrospective study, paravertebral block was found to be feasible for providing intraoperative anesthesia for percutaneous nephrolithotomy. Three patients experienced severe discomfort and bladder irritation during ureteroscopy; however, the combination of a low dose of propofol and to pical lidocaine gel met the surgical requirements. Since the failure rate of paravertebral block for percutaneous nephrolithotomy in some reports was 6.1–20% (27, 28), VAS scores were assessed after paravertebral block treatment. Analgesia was given to patients with VAS > 3 to ensure their comfort. In our study, the average VAS pain score was <2 in PVB group, suggesting that paravertebral block can meet the operation needs of most patients, except for two patients who were administered remifentanil because the blocking effect was incomplete. Since six-segment injections were applied rather than a single one (29), the failure rate in our study was lower than those of previous reports. All patients, except those who were administered propofol, were awake and satisfied with the level of anesthesia, indicating that this technique is effective and meets the surgery requirements. Achieving adequate analgesia for percutaneous nephrolithotomy requires the blockage of skin and muscle innervation and visceral nerves for the kidney and ureter (30). The tract to block for lithotripsy typically includes the 10th and 11th intercostal spaces (31). Sensory nerves in this area are readily anesthetized by a thoracic paravertebral block, while kidney and ureter nerves originate from T_10_ to L_1_ (13, 25). In contrast to previous reports (26), we observed that blocking the homolateral spinal nerves from T_8_ to L_1_ led to satisfying sensation reduction and provided effective anesthesia for percutaneous nephrolithotomy.

In our study, there were no significant differences in terms of age, sex, weight, height, and comorbidities between the patients who underwent paravertebral block and general anesthesia. Owing to the aging population in Shanghai (32), the patients treated at our hospital were mainly elderly, although the typical onset age of calculi is in young adulthood (3); the mean age of the patients in our study was >60 years. In elderly patients, the physical conditions often decay (33). The PVB group had higher ASA and heart function scores, including several patients with ASA IV, who were at high risk of receiving general anesthesia; however, nephrolithiasis pain is severe and must be treated promptly and effectively (34). Paravertebral block affects circulation and respiration less than general anesthesia and can be administered to high-risk patients in poor general conditions or with several underlying diseases, in contrast to general or neuraxial anesthesia. Several patients still required sedatives or analgesics, which might have increased the anesthesia risks. However, the small doses of sedatives or analgesics had minimal effect on circulation and respiration (35, 36). Moreover, the percentage of patients requiring propofol or remifentanil was low; all ASA III–IV patients in our study received only the paravertebral block, without additional sedatives or analgesics. The incidence of intraoperative hypotension, a risk factor for malignant cardiovascular and cerebrovascular events (37), was significantly lower in the PVB group compared with the GA group. There was only one patient that had oxygenation decline during surgery in the PVB group due to the additional sedatives or analgesics (38). However, the hypoxemia was promptly corrected with effective treatment. In addition, potential complications of paravertebral block, including bleeding and nerve damage, were not identified. Overall, the incidence of adverse events was low in the PVB group, and this technique was deemed safe for percutaneous nephrolithotomy.

Furthermore, there were no significant differences between groups regarding the surgical data. The anesthesia duration was longer in the PVB group since ultrasound-guided nerve block requires more time to be fully effective. Other differences included the volume of infused fluids, as the patients often needed extra fluids under general anesthesia due to peripheral vasodilation. No significant differences were found in hospitalization and anesthesia satisfaction in both surgeons and patients. The overall cost for the paravertebral block was significantly lower than for general anesthesia, alleviating the economic burden for the patients. Overall, the paravertebral block was similar to general anesthesia in ensuring surgical safety and patient comfort at a lower cost.

As some of the patients in the GA group received PCIA, we compared the postoperative data of the three groups. We found that the incidence of nausea and vomiting was significantly higher in patients who underwent general anesthesia than in the paravertebral block group. Postoperative analgesia had no significant effect on nausea and vomiting in the two general anesthesia groups. Although we did not analysis postoperative throat pain and hoarseness caused by intubation, or adverse reactions such as slow peristalsis and hypothermia caused by general anesthesia, the use of paravertebral block obviously prevented them from occurrence.

Regarding postoperative pain, the scores of the paravertebral block and GA-2 group (with PCIA) were lower than that in the GA-1 group (without PCIA) immediately after surgery. However, after 6 h, the VAS score in paravertebral block patients increased due to the gradual decay of the blocking effect, approaching the pain score of the general anesthesia patients without PCIA (21). The patients with PCIA had a lower VAS score than those in the other two groups at the 24-h time point. Nonetheless, the mean VAS scores of the three groups at this time were <2, suggesting that the postoperative pain was generally limited to the first day after surgery; this finding is consistent with previous reports (13, 23). There was no significant difference in pain scores among the three groups 48 h after surgery. Overall, paravertebral block was more effective than general anesthesia alone in relieving postoperative pain, although it does not play a continuous analgesic role.

There are some limitations both to this study and the application of paravertebral block. First, retrospective studies are prone to data bias, and the intra- and postoperative data were incomplete. Second, VAS scores may be unreliable due to the subjective nature of experiencing and reporting pain (13). Additionally, since paravertebral block is unilateral, patients may feel uncomfortable after bladder flush and expansion during ureteroscopy. Bladder irritation signs are evident after ureter catheter placement; thus, additional sedative and analgesic drugs are sometimes needed. In future clinical practice and research, optimizing this anesthesia method to ensure safe and comfortable operating conditions is necessary. Furthermore, we retrospectively compared the effects of paravertebral block and general anesthesia; however, a prospective randomized controlled trial should be conducted.

In this retrospective study, paravertebral block was effective and safe in providing intraoperative anesthesia for percutaneous nephrolithotomy, and had less adverse events and anesthesia costs. Paravertebral block is an attractive alternative for patients at increased risk of comorbidities following general or neuraxial anesthesia.

## Data availability statement

The original contributions presented in this study are included in the article/supplementary material, further inquiries can be directed to the corresponding authors.

## Ethics statement

The studies involving human participants were reviewed and approved by the Institutional Research Ethics Committee of the Shanghai Fourth People’s Hospital (approval number: 202011115-001). Written informed consent for participation was not required for this study in accordance with the national legislation and the institutional requirements.

## Author contributions

LX, CL, MF, and WQ: substantial contribution to conception and design. GA, DL, and MF: analysis and interpretation of data. MF and CL: writing manuscript. All authors: acquisition of data and revising manuscript.
